# Reconciling competence and transcriptional hierarchies with stochasticity in retinal lineages^☆^

**DOI:** 10.1016/j.conb.2014.02.014

**Published:** 2014-08

**Authors:** Henrik Boije, Ryan B MacDonald, William A Harris

**Affiliations:** Department of Physiology, Development and Neuroscience, Cambridge University, Cambridge, UK

## Abstract

Recent advances suggest that there is a stochastic contribution to the proliferation and fate choice of retinal progenitors. How does this stochasticity fit with the progression of temporal competence and the transcriptional hierarchies that also influence cell division and cell fate in the developing retina? Where may stochasticity arise in the system and how do we make progress in this field when we may never fully explain the behavior of individual progenitor cells?

## Highlights

• Problems with a strict retinal competence model are explained.
• The apparent conflict between transcriptional hierarchies and stochasticity is resolved.
• The underlying nature of retinal progenitor cell stochasticity is discussed.
• Key issues that can be addressed in the face of stochasticity are enumerated.

**Current Opinion in Neurobiology** 2014, **27**:68–74

This review comes from a themed issue on **Development and regeneration**

Edited by **Oscar O Marín** and **Frank F Bradke**

For a complete overview see the Issue and the Editorial

Available online 15th March 2014

0959-4388/$ – see front matter, © 2014 The Authors. Published by Elsevier Ltd. All rights reserved.

**http://dx.doi.org/10.1016/j.conb.2014.02.014**

## The conflict

**Figure 1:**
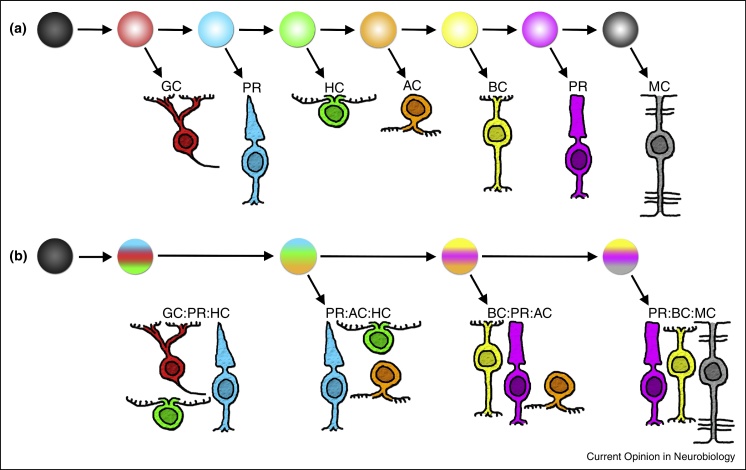
The competence model. The classical view sees retinal progenitor cells progressing through competence windows during which a particular cell type is generated **(a)**. Recent studies suggest that although a unidirectional transition of competence occurs, progenitor cells choose from multiple fates at any one time **(b)**.

More than two decades ago, clonal analysis in the retina revealed the multipotency of retinal progenitor cells (RPCs) [1–3]. The widely accepted competence model proposed by Livesey and Cepko [4] put multipotency into the context of the previously described evolutionarily conserved order of retinal histogenesis correlated to the fact that clones generated early, produce both early and late generated cell types, while clones generated later produce only late cell types [5,6]. The competence model suggests that RPCs acquire and then lose the ability to make various cell types as retinal development proceeds (Figure 1a). It was proposed that the progression of competence might be largely regulated by extrinsic signalling — that instructive environmental cues could be changing as a function of development [5,7]. However, no convincing instructive cues have been found. Indeed, cell-mixing and transplant experiments revealed that young RPCs in older environments do not change their temporally appropriate fates [8–10]. More recently, it was shown that RPCs grown in isolation give rise to clones that are similar both in size and composition to clones *in vivo* [11^•^,12^••^]. Thus, a changing external environment is neither essential, nor adequate, to achieve histogenetically appropriate fates (although it should be noted that environmental cues may nevertheless provide negative feedback to fine-tune the proportions of cells that acquire particular fates [13–15]). The competence model must therefore rely on an intrinsic progression in fate potential. Indeed, the intrinsic nature of cellular diversification in the developing retina is consistent with a large and growing literature on various of transcription factors (TFs), often working together within hierarchies, that are involved in specifying cell fates [16,17].

A puzzling aspect of retinal development in light of these transcriptional cascades has come from recent theoretical treatments of the statistical properties of retinal clones, which are variable in cell number and fate composition. This work shows that the variability of cell number among clones can be accurately accounted for by assuming that RPCs are equipotent and their proliferation is in part stochastic [12^••^,18^•^,19^••^]. This work also shows that cell fate variability among clones is likely to have a partially stochastic explanation [12^••^,19^••^]. The fact that proliferation and fate might be in part stochastic does not mean that these processes are uncontrolled, random or unregulated, but rather that they operate according to defined probabilities and predictable ensemble behaviors that are statistically well behaved. Consistent with the predictions of these stochastic models, live imaging studies have shown that the daughters of individual RPCs do not appear to obey a strict temporal program of fates. Rather they sometimes give rise to cell types within a clone that are reversed in their order of appearance to the overall order of histogenesis, and are thus contrary to the predictions of a strict competence model [12^••^,19^••^,20] (Figure 1b). These findings raise questions about how our understanding of intrinsic progression of RPCs, and TF hierarchies, can be reconciled with the stochastic nature of clonal lineages.

## The transcriptional circuitry of retinal cell fate

**Figure 2:**
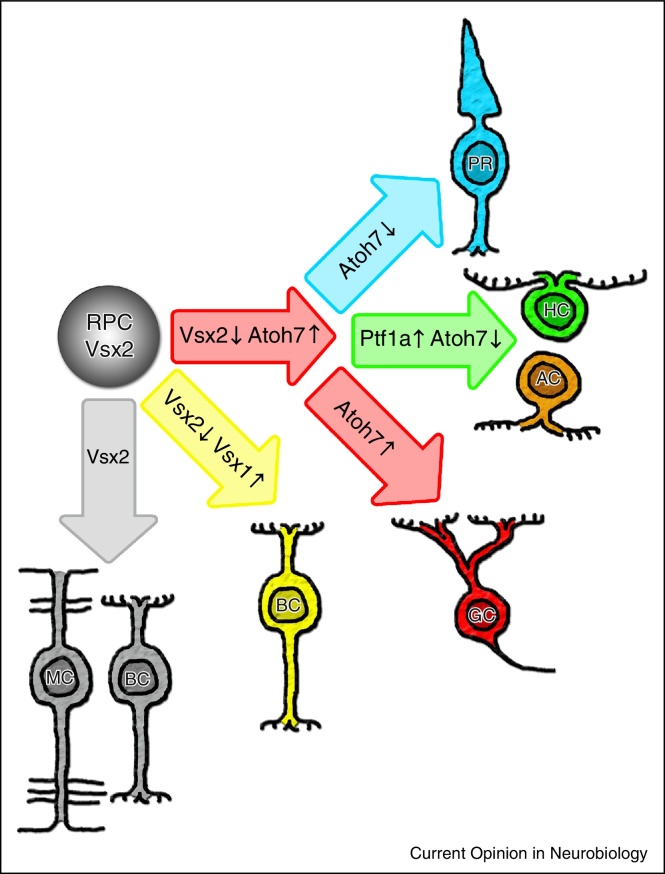
Core transcriptional hierarchies. During the early proliferative phase of retinal development all RPCs express Vsx2, which inhibit factors such as Atoh7 and Vsx1. As development progress this inhibition is abolished and genes influencing cell fate are expressed. Depending on the level of Atoh7, and presence or not of Ptf1a, the progenitor follows different paths giving rise to different cell fates. The Vsx1-lineage gives rise to a distinct population of BCs from the population expressing Vsx2.

It is clear that numerous TFs expressed in RPCs play roles in the specification of retinal cell types. In a number of vertebrates, there is a core transcriptional hierarchy (Figure 2), which can explain some of the molecular decisions that retinal cells must make to achieve particular fates. The TF Atoh7 is required for the generation of GCs [21,22] and prevents PR fate by inhibiting genes required for their development [21,23]. Loss of Atoh7 leads to an increase in cone PRs suggesting that the absence of Atoh7 provides a permissive environment for a fate shift to cones [24]. Ptf1a can inhibit Atoh7 expression and is necessary for the specification of HCs and ACs [25,26]. Misexpression of Ptf1a causes an increase in HCs and ACs at the expense of GCs, PRs and BCs indicating that Ptf1a is sufficient for the re-specification of these cell types [20,26]. Vsx2 is initially expressed throughout the RPC pool, and represses the expression of Atoh7, FoxN4 (an upstream regulator of Ptf1a expression) and Vsx1 [27^•^,28]. Vsx2 is down-regulated in all but a small population of RPCs that will give rise to a subset of BCs and MCs. The Vsx1-lineage gives rise to a subset of BCs distinct from the Vsx2-lineage derived BCs [27^•^]. Loss of FoxN4 or Ptf1a prevents HC genesis, severely reduces the number of ACs, and leads to an increase of PRs and GCs [25,29,30].

**Figure 3:**
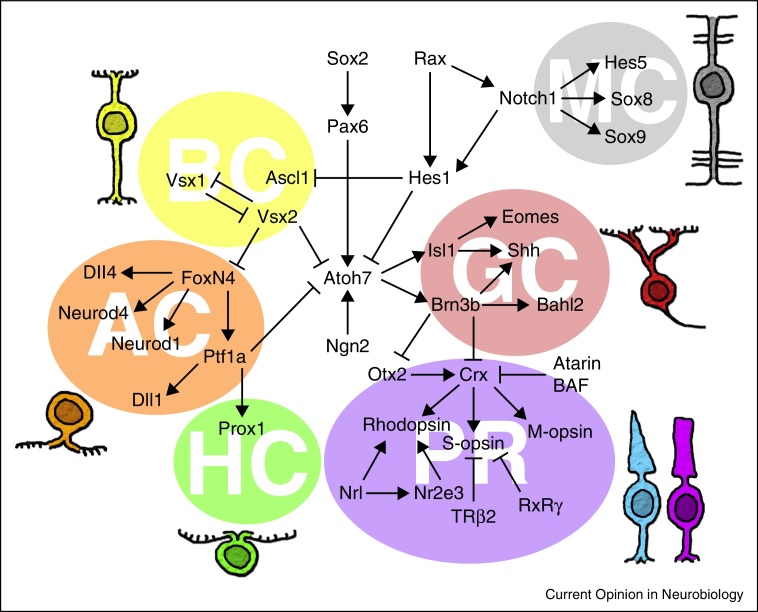
The complexity of transcriptional regulation. Although key factors can explain some the diversification there are numerous factors affecting fate outcome. Cross-talk between branches increase the complexity of the system.

While this core hierarchy may explain how the major cell types arise, a number of studies, too many to review here, have revealed that several additional factors that also influence particular retinal fates (Figure 3, revised from [31]). Moreover, the simultaneous expression of two or more TFs can synergistically influence fate suggesting that combinatorial coding also plays an influential role cell fate diversification [32–34]. From these studies, it appears that the intrinsic core hierarchy of retinal cell determination is overlaid with a complex weave of transcriptional circuitry that makes it challenging to predict which cell types will arise from particular progenitors.

Many of the TFs discussed above are expressed only when cells exit or are about to exit the cell cycle and seem to act by specifying one fate over another. In other words, most of them control what the daughter cells of RPCs will become once they exit the cycle but not the competence of RPCs. Competence controlling factors should be expressed in dividing RPCs during the time that they are making particular cell types. They should also act upstream of the fate determining genes, perhaps by increasing the chance that particular sets of these fate determining genes are turned on or off. Such temporal competence factors are clearly seen in Drosophila CNS neuroblasts, where a sequence of fate-influencing TFs starting with Hunchback are expressed [35]. There is some evidence for similar temporal competence factors playing a role in vertebrate neurogenesis. For example, in the mouse, RPCs pass through an early stage in which they express Ikaros, the vertebrate orthologue of Hunchback. Ikaros, when overexpressed, biases the production of early fates, while Ikaros mutant mice have reduced numbers of early-born cell types [36^•^,37]. One cannot rule out the possibility that an entire sequence of competence factors homologous to those found in Drosophila neuorblasts will be found in the vertebrate retina, but at present there is scant evidence for this.

## Clonal stochasticity

**Figure 4:**
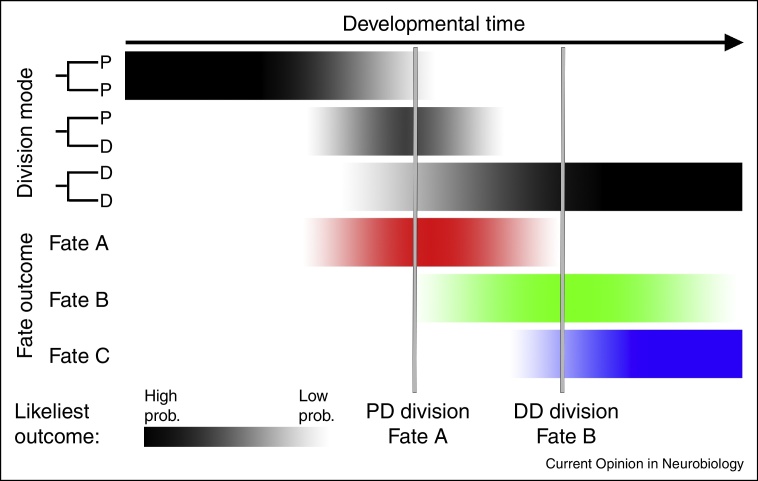
Reconciliation. Stochastic models can predict the proliferative properties of RPCs, whether the daughter cells of RPCs continue to proliferate (P) or differentiate (D). In a similar way fate may be assigned in a stochastic way within a progression changing probabilities.

The statistical distribution of clone sizes seen in both late rat RPCs *in vitro* and zebrafish RPCs *in vivo* fits well with a model that assumes RPCs are equipotent but that the mode of division (proliferative (PP) versus asymmetric (PD) versus differentiative (DD)) is stochastic [12^••^,19^••^]. Layered on top of this stochasticity, however, is a progressive program in which the probability for particular modes of division ontogenetically evolves. For example, in the zebrafish retina at early stages, all divisions are proliferative. This is followed by a period where each division mode (PP, PD and DD) occurs with approximately equal probability. The final stage of retinal proliferation is another stochastic period dominated by DD divisions (Figure 4) [19^••^]. This simple model not only accounts for the distribution of clone sizes from RPCs at different stages of development, it also accurately predicts division patterns observed in a population of individual RPCs *in vivo*.

The choice of fate also appears to have a stochastic element. Gomes *et al.* [12^••^], found that the cell fates in more than one hundred clones from a rat retina were largely consistent with the hypothesis that these late progenitors were equipotent but choosing their fates stochastically, with the relative possibility for each cell type being equivalent to the proportions of these cells in the mature retina. However, it has to be said that a few combinations of fate within clones appear more or less frequently than expected, indicating that in addition to the overriding stochasticity, there may also be some preprogrammed motifs operating according to underlying, but as yet unknown, rules. For example, it was recently found that a subset of RPCs express the TF Olig2 and were biased toward production of rod PRs and ACs [38]. Similarly, GCs that respond to vertical motion arise from progenitors that express Cdh6 [39]. In the zebrafish analysis [19^••^], there were also some patterns that could not be explained by a stochastic mechanism, such as the fact that at late stages of retinogenesis, most PRs, BCs and HCs come in pairs. All of these instances, however, may reflect the action of TFs operating very close to the last division to specify particular fates. Asymmetrically inherited Numb may also be at play here. If, for example, Numb, is inherited by one of the two daughters at a terminal division, the two daughter will chose two different fates (e.g. a dominant fate taken by the Numb inheriting cells and a secondary fate taken by the other). This could explain why some terminal divisions are partially patterned, though it may be impossible to predict in advance of the division which daughter will inherit Numb and thus which daughter will take which fate [40].

## Why stochasticity?

It is interesting to speculate about the mechanisms that generate stochasticity within retinal lineages. We can imagine that levels of TFs themselves might be variable, due to dynamic changes in transcription rate, translation efficiency, or mRNA and protein stability (as reviewed in [41]). There may even be mechanisms for generating a stochastic outcome. For example, the choice of red versus green opsin in the primate retina relies on the random looping of DNA to bring a single promoter region adjacent to one of the two protein coding regions [42,43]. Variability may also arise through post-transcriptional mechanisms involving mi-RNAs and long noncoding RNAs, or post-translational mechanisms such protein phosphorylation and ubiquitination through interaction with cell cycle enzymes [44,45]. It is also likely that epigenetics, the packing and remodeling of chromatin in the nucleus, will affect the chance that a specific locus will fire or not [46].

The Notch-Delta signaling mechanism may also contribute to the stochastic decisions that RPCs make [47–49]. This mechanism can magnify small fluctuations in fate potential and may also lead to oscillations. Indeed the expression of the Notch downstream target, Hes1, is known to oscillate at rates much shorter than the minimal cell cycle time in neural progenitor cells in culture, and pairs of interacting cells may oscillate out of phase with each other [50]. The pattern generator for this rhythm may lie within individual progenitors due to a cell intrinsic double negative feedback loop in which miR-9 controls the stability of Hes1 mRNA, while Hes1 represses the transcription of miR-9 [51]. Recent studies in the mouse telencephalon have shown that proneural TFs also oscillate in progenitor cells possibly in response to the oscillations of the Hes1 repressor [52]. Interkinetic nuclear migration along the apico-basal axis of the neuroepithelium may also contribute to stochasticity through this pathway. For example, Notch signaling tends to be apical, and cells whose nuclei are more apical may be influenced to a greater extent [53]. But as the apico-basal movements of RPC nuclei throughout most of interphase are themselves stochastic [54], the efficacy of Notch signaling could be affected by this random one-dimensional walk. Similarly, as mentioned above, the asymmetric inheritance at the last division of Numb, a negative regulator of Notch signaling, may contribute to stochasticity by influencing which daughter cell which choose a dominant fate and which will choose a secondary one [39].

Finally, it is unknown to what extent the multiple transcriptional hierarchies present within RPCs interact. In the face of combinatorial coding mechanisms where different TFs have non-additive influences on fate choice, asynchronous, loosely coupled, or independently firing networks could mean that such combinations of TFs may appear probabilistically within single PRCs. All of these stochasticity-generating mechanisms may be going on simultaneously within RPCs, suggesting that a high level of uncertainty is inherent in this system. This is not necessarily a bad thing. Complex systems in which many variables interact often produce robust and well-behaved distributions such as the relative proportions of ‘snake eyes’ versus ‘lucky sevens’ in a large population of dice throws. Similarly, although individual RPCs give rise to clones that are highly variable, the total number of differentiated retinal cells generated from the 2000 or so RPCs of the zebrafish optic vesicle will always be very close to 22 000, and within this large set of differentiated retinal cells, all the major neuronal types will proportionally represented [19^••^].

## Research after reconciliation

Stochasticity can be seen as a problem. It may be disappointing to think that we may never be able to predict exactly what a set of RPCs will do; which cells will divide how many times and what the fate outcomes of these divisions will be. But while this kind of stochasticity is like a cloud that obscures the answers to certain questions, it is a cloud that has a silver lining, in that it focuses our attention on other questions that may be easier to address, and even perhaps more interesting. For example, recent studies show that eliminating certain TFs leads to fate switches in daughter cells rather than the death of particular cell types. As a result, such retinas may have vastly altered cell fate distributions while the number of cells in such retinas may be very similar to wild type retinas. Such results suggest that proliferation and fate may therefore be best explained by independent and largely uncoupled stochastic mechanisms, and this makes sense as many of the TFs that have major roles in cell fate are not expressed until cells are about to leave or have just left the cell cycle. Another important issue is that, in spite of the stochastic noise, retinal development clearly progresses through distinct phases of proliferation and cell fate probabilities (i.e. at each stage of development we can accurately predict the population distributions of proliferative/differentiative divisions and the cell fate distributions). Clearly, the next step is to understand what it is that determines the transition between these phases. What is the timer and how does it work? We would also like to know more about how the probability profiles at each phase are themselves controlled. In the developing retinas of some animals, for example, it is likely that probability of asymmetrical divisions during the middle phases will be higher than in other animals, or the probability of rods may be much higher than the probability of cones. What are the factors that set these probabilities and are they the same factors that are at the heart of the evolution of retinal size and cellular composition within vertebrates? Finally, we would like to know more about the extrinsic versus intrinsic influences on cell proliferation and fate. For example, do large clones tend to have small clones as neighbors, or are the decisions that are made within each clone independent of the behavior of neighboring clones? Therefore, crucially, while the mechanisms that generate stochasticity are interesting to consider, it may be more productive to investigate those features of retinal development that are independent of stochasticity and remain salient in spite of it. Lastly, it will be useful to know if the concepts outlined here for the retina are also applicable to other parts of the nervous system, or even other tissues.

## References and recommended reading

Papers of particular interest, published within the period of review, have been highlighted as:

• of special interest
•• of outstanding interest
